# Integrated single cell transcriptomics analysis for unraveling heterogeneity and plasticity of root cells for sustainable and regenerative agriculture

**DOI:** 10.3389/fpls.2026.1881910

**Published:** 2026-09-07

**Authors:** Erum Yasmeen, Muhammad Riaz, Bilal Saleem, Ghalia Alameri, Mayank Anand Gururani

**Affiliations:** 1 Single Cell Research Center, Department of Plant Sciences, School of Agriculture and Biology, Shanghai Jiao Tong University, Shanghai, China; 2 Institute of Plant Nutrition and Environmental Resources, Beijing Academy of Agriculture and Forestry Sciences, Beijing, China; 3 Beijing Engineering Technology Research Center for Slow/Controlled-release Fertilizer, Beijing, China; 4 National Institute of Genomics and Advance Biotechnology, National Agriculture Research Centre, Islamabad, Pakistan; 5 Department of Chemistry, College of Science, United Arab Emirates University, Al Ain, United Arab Emirates; 6 Biology Department, College of Science, United Arab Emirates University, Al Ain, United Arab Emirates

**Keywords:** root system architecture (RSA), regulatory networks, transcriptional landscape, single-cell RNA sequencing, regenerative agriculture

## Abstract

The plant root system architecture (RSA) functions in anchorage, acquisition of water and mineral nutrients, and exhibits pronounced phenotypic plasticity in response to the spatiotemporal heterogeneity of the soil environment. Resolving the regulatory networks that underpin root development is therefore a prerequisite for improving stress tolerance and yield. Single-cell RNA sequencing (scRNA-seq) resolves transcriptional landscapes at cellular resolution, discriminating root zonation, lineage trajectories and cell-type-restricted responses to environmental signals. Coupling scRNA-seq to epigenomic, proteomic and metabolomic profiling of the same cell populations links chromatin state to transcript, protein and metabolite output and therefore exposes the regulatory layers that govern root development and plasticity. Machine-learning models trained on single-cell matrices assist cell-type annotation, gene regulatory network inference and prioritization of candidate loci for precision breeding, although their output remains contingent on reference datasets that are still sparse for crop species. This review examines what scRNA-seq, spatial transcriptomics and machine learning have so far established about root cellular heterogeneity and regulatory architecture. This delimits the technical constraints that presently bound their application to crop improvement including protoplasting bias, transcript dropout and incomplete state of crop reference atlases to support sustainable and regenerative agriculture.

## 1 Introduction

**Figure 1:**
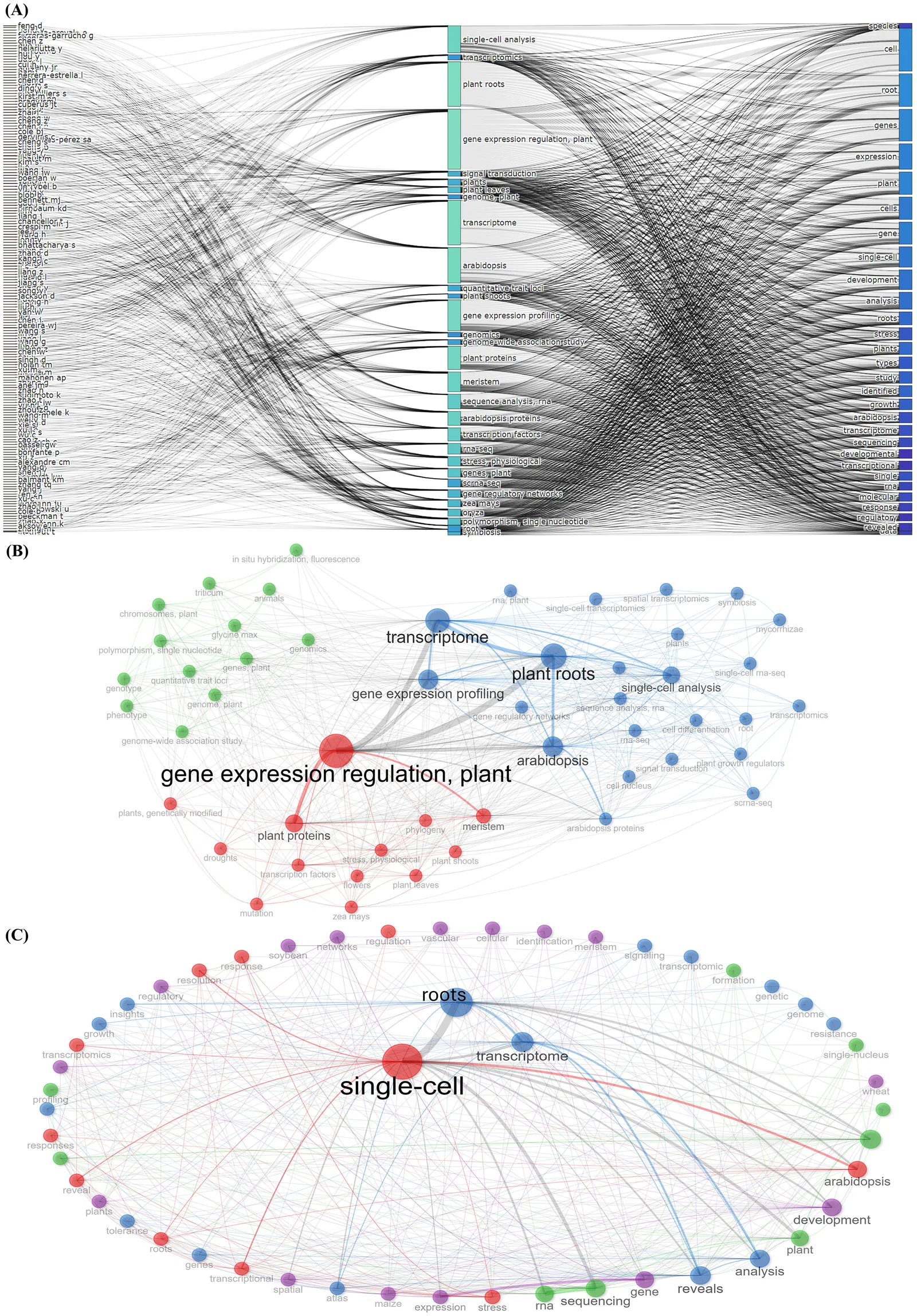
Bibliometric visualization of plant root single cell transcriptomics research findings. **(A)** Sankey diagram of the co-occurrence relationships linking the most productive authors, author keywords and indexed keywords across the field. **(B)** Co-occurrence keyword’s network clustering in gene expression regulation in plants (red), transcriptome and single-cell analysis (blue) and genomic/genetic variation (green) with node size representing keyword occurrence and edge thickness proportional to co-occurrence frequency. **(C)** Thematic or central keywords representing “single cell” and “transcriptome” with connection to roots and sub-clusters spanning root biology, spatial transcriptomics, stress responses and comparative genomics across plant species.

Terrestrial plants are the outcome of prolonged selection for resource capture under competition. The root, the principal below-ground organ, is functionally versatile and central to that capacity (Lin et al., 2024; Sharma et al., 2025). The root anchors the plant against gravitational and mechanical load. It is also a center of structural, physiological and biochemical activity that determines plant survival and productivity (Pereira et al., 2024). Roots acquire water and soluble nutrients from spatially heterogeneous soil, and synthesize and export the growth regulators that coordinate development of the aerial tissues (Gregory, 2007). At the soil interface roots both register environmental cues and establish symbioses with soil microorganisms, jointly determining soil function and plant performance (Akhtar and Hakeem, 2025). Roots further contribute to ecosystem function through exudation of carboxylates and other low-molecular-weight compounds, which govern organic matter turnover and structure the rhizosphere microbial community that controls nutrient availability (Bolan et al., 2025). Root system architecture is developmentally plastic, permitting growth to be redirected in response to the physical and chemical heterogeneity of the soil (Schneider and Lynch, 2020). This plasticity rests on pronounced cellular heterogeneity, itself generated by the developmental programs that specify lineage identity, positional information and differentiation state. These regulatory circuits and their relation to radial and longitudinal patterning have been progressively resolved over the past two decades (Petricka et al., 2012; Birnbaum, 2018). A bibliometric survey was performed in Biblioshiny (Bibliometrix R package) retrieved 368 records from the Web of Science and PubMed central collection using the query “root single-cell” or “plant root single cell transcriptomic” restricted to articles and reviews published between year 2016 and 2026 (**Supplementary File 1**). The duplicate records were removed and keyword variants merged before co-occurrence mapping (**Figure 1**) (Aria and Cuccurullo, 2017). Gaps nonetheless remain in the molecular description of cell fate acquisition and of how root cells register and respond to physical constraints such as soil compaction (Birnbaum et al., 2003; Ogorek et al., 2025). Root biology has historically been conducted on whole organs or bulk tissue. Such measurements average across cell populations and therefore cannot assign a transcriptional response to the lineage in which it arises. Single-cell RNA sequencing was first reported in 2009 by Tang et al., 2009, (Tang et al., 2009) on a single mouse blastomere, establishing that cell-to-cell transcriptional variation could be measured directly. Cell-type-resolved transcriptomics in plants followed, with Efroni et al., 2015 and 2016, introducing a quantitative index of cell identity from single-cell expression profiles in the *Arabidopsis* root (Efroni et al., 2015, 2016). The droplet-based scRNA-seq of the intact Arabidopsis root was reported independently in 2019 (Denyer et al., 2019; Ryu et al., 2019). The scRNA-seq resolves root development by profiling the transcriptome of individual cells, exposing cell state, differentiation stage and the transcriptional relationships between them (Han et al., 2024). Coupled to complementary single-cell assays it also reports chromatin accessibility and transcript dynamics within defined cell populations, from which the cellular hierarchy and regulatory architecture of the root can be reconstructed. It does not, however, track biomolecular transport or subcellular localization, which require orthogonal imaging approaches. scRNA-seq has revised how root architecture and function are interpreted, chiefly by locating the regulatory nodes that govern development and adaptive capacity within individual cell types (Shahan et al., 2022; Zhao et al., 2024). These findings supply candidate targets for breeding tolerance to abiotic and biotic stress. It should be noted that scRNA-seq yields a fixed snapshot of the transcriptome at the moment of tissue dissociation and does not report physiology in real time. This review assesses what scRNA-seq, its multi-omic extensions and machine-learning analysis have established about root regulatory mechanisms and genome dynamics, and identifies where the evidence remains provisional. Single-cell measurement combined with computational inference addresses long-standing gaps in the functional description of crop roots. Improved resolution of root biology is a prerequisite for cultivars with greater nutrient-acquisition efficiency and lower input demand supporting regenerative agriculture (Lynch, 2013).

## 2 scRNA-seq resolves root cellular heterogeneity, growth and the developmental landscape

**Figure 2:**
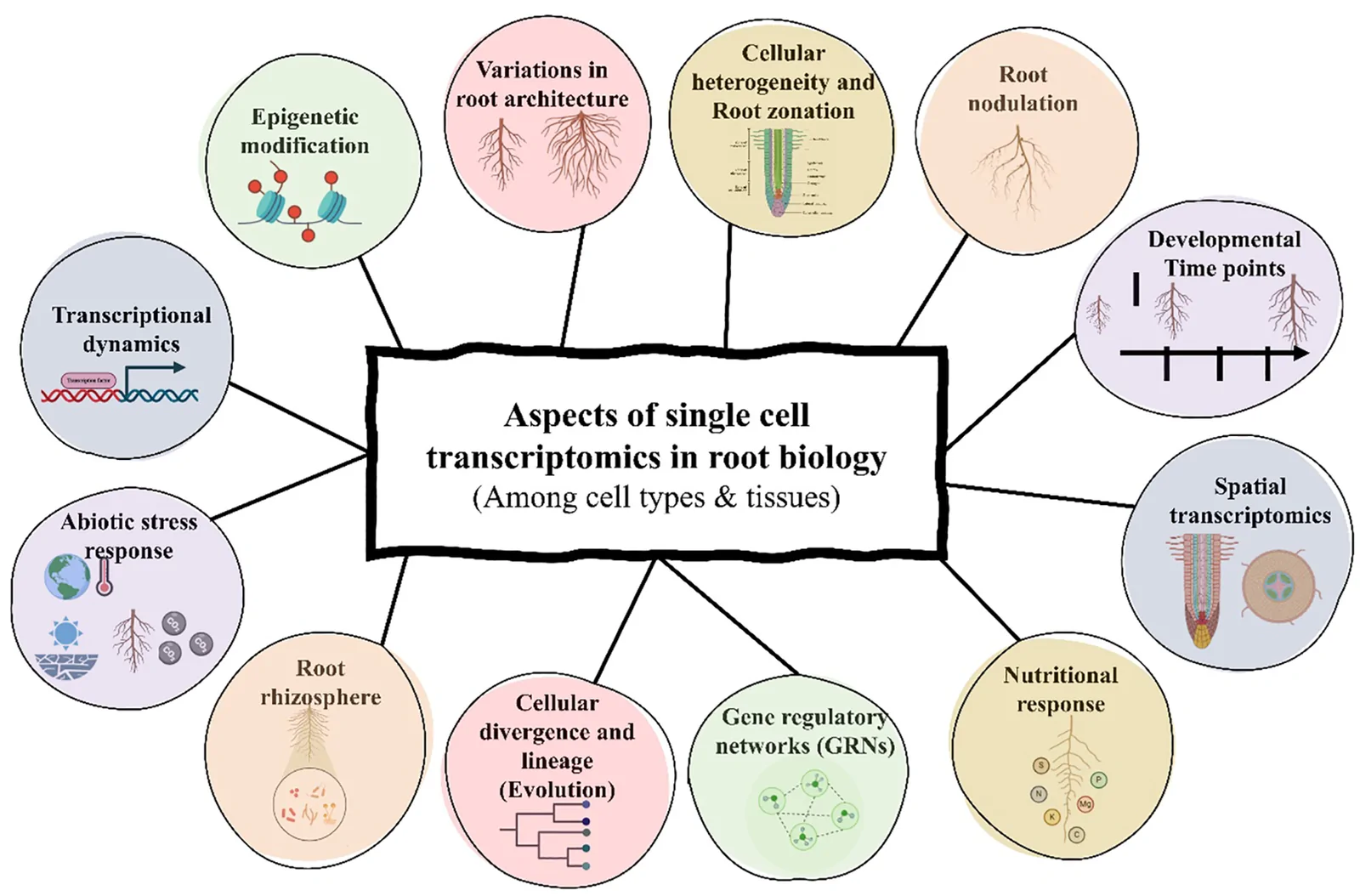
Single-cell transcriptomics decoding plant root biology. scRNA-seq resolves root cellular heterogeneity, zonation, transcriptional dynamics, epigenetic regulation, lineage divergence and GRNs. Spatial transcriptomics localizes these expression patterns across developmental time points and stress responses.

The root exhibits extensive cellular heterogeneity at every developmental stage, comprising specialized cell types with defined roles in root function and development (Yin et al., 2024). Root development and elongation involve coordinated cellular processes operating at single-cell resolution (**Figure 2**). The primary root is organized longitudinally into four functionally distinct zones: the meristematic zone (MZ), the transition zone (TZ), the elongation zone (EZ) and the maturation or differentiation zone (DZ) (Verbelen et al., 2006). The MZ, also termed the root apical meristem (RAM), lies at the root apex and harbors the stem cell niche (SCN) (Han et al., 2024). The SCN comprises mitotically quiescent cells at the distal end of the RAM, surrounded by pluripotent stem cell initials. Indeterminate root growth is maintained by the SCN, whose activity is regulated by the quiescent center (QC) and adjacent stem cell initials. Under QC-mediated signaling, stem cell initials undergo successive asymmetric divisions, generating daughter cells that acquire specialized identities as they pass through elongation and differentiation (Gregory, 2007). These cell populations are organized into concentric, radially symmetric layers comprising the epidermis, cortex, endodermis, pericycle and vascular tissues. Shootward of the MZ, the TZ constitutes a developmental boundary at which cells exit the proliferative program and undergo cytoskeletal reorganization before entering elongation; it also serves as a principal site of environmental signal perception (Jean-Baptiste et al., 2019). Cell expansion then occurs predominantly in the EZ, where cells enlarge anisotropically without further division. Wall loosening through modification of the structural polysaccharides cellulose, hemicellulose and pectin permits sustained longitudinal elongation. In the DZ elongation ceases and cells acquire their terminal identities (Brady et al., 2007; Goldy et al., 2025). These structural and functional specializations are prerequisites for nutrient acquisition, mechanical support and whole-plant growth (Han et al., 2024).

### 2.1 Single-cell platforms, analytical pipelines, and root cell atlases

Single-cell RNA sequencing (scRNA-seq) has advanced the description of root cellular heterogeneity, resolving gene expression at single-cell level, classification of cell populations, and identification of cell-type-specific marker genes in the primary root (Shahan et al., 2021). High-resolution profiling of heterogeneous root cell populations now draws on droplet-based scRNA-seq and snRNA-seq (Zheng et al., 2017) and on spatial platforms including Visium (Ståhl et al., 2016) and imaging-based MERFISH/seqFISH (Chen et al., 2015; He and Pryhuber, 2025). It should be noted that the imaging-based methods were developed for animal tissue and remain sparsely applied to crop roots, where cell wall autofluorescence and probe penetration are limiting. The computational pipelines FastQC (Zhang and Kang, 2021), STAR (Dobin et al., 2013) and Cell Ranger (Zheng et al., 2017) support quality control and read alignment, while Seurat and Scanpy handle pre-processing, clustering and cell-type annotation (Lähnemann et al., 2020). Datasets from different experiments are harmonized by batch-effect correction with Harmony or scVI (Korsunsky et al., 2019) and developmental dynamics and tissue organization are recovered by trajectory inference and spatial mapping in Monocle, scVelo and Tangram (Trapnell et al., 2017; Bergen et al., 2020; Biancalani et al., 2021). Together these steps constitute the analytical pipeline summarized in **Table 1**. While cell identity may remain stable, individual cells respond dynamically to changing physiological and developmental signals. scRNA-seq therefore provides a direct approach to the context-dependent behavior of individual cells (Oliva et al., 2022). The arabidopsis root scRNA-seq resolved 4,727 cells and approximately 16,975 genes partitioned into discrete expression clusters (Zhang T. Q. et al., 2019) and subsequent organ-scale atlases raised this by more than an order of magnitude (Shahan et al., 2022). The clusters recovered lineage relationships among cell and tissue types and resolved spatiotemporal developmental states. Ryu et al., 2019, demonstrated the utility of scRNA-seq in resolving rare cell subpopulations in the *Arabidopsis thaliana* root tip and characterizing their transcriptional signatures, with immature meristematic cells concentrated at the root apex and differentiated cells distributed shootward and peripherally (Ryu et al., 2019). This spatial organization underscores the pronounced cellular heterogeneity of root tissues, with each cluster exhibiting a distinct gene expression program reflecting progressive differentiation and positional identity (Zhang T. Q. et al., 2019). For example, root-hair and non-hair expression signatures in meristematic cells permit lineage mapping of epidermal cell types, whereas QC identity is delineated subtractively, by the absence of epidermal and other tissue-specific markers (Wendrich et al., 2020). Liu et al., 2021, extended scRNA-seq to the monocot *Oryza sativa*, profiling over 20,000 cells and identifying cell populations without an Arabidopsis counterpart (Liu et al., 2021). In addition, the regulatory networks underlying cell fate determinations were elucidated through dual rice root atlases integrating chromatin accessibility profiling and inter-species comparative analysis (Zhang et al., 2021b). Representative root single-cell studies are collated in **Table 2**. Pseudotime analysis reconstructs differentiation trajectories and quantifies gene expression variance across cell states, resolving rare populations and providing molecular definition of root zonation (Denyer et al., 2019). Yin et al., 2024, used time-course scRNA-seq to resolve cellular heterogeneity and cell fate transitions during early callus formation from Arabidopsis root explants (Yin et al., 2024). Integration of temporal and spatial transcriptomic data extends this to the tissue scale, and pan-transcriptome resources for agricultural species permit cell identities and differentiation programs to be compared across crops (Giacomello et al., 2017; Nolan and Shahan, 2023; Ke et al., 2025). The study by Guillotin et al., 2023, compared three grasses, *Zea mays*, *Sorghum bicolor* and *Setaria viridis*, by scRNA-seq and snRNA-seq, examining the consequences of whole-genome duplication, identifying rapidly evolving cell types and describing the modular genetic architecture underlying agronomic traits (Guillotin et al., 2023). Co-expression network analysis of scRNA-seq data identified a module of 149 genes accounting for the predominant expression program across *Arabidopsis thaliana* root developmental stages (Han et al., 2024). A root phloem cell atlas resolved phloem pole identity with positional accuracy and identified *PAPL genes* as mediators of long-distance sugar transport to the root (Otero et al., 2022).

**Table 1:** Integrated pipeline for crop root single-cell and spatial transcriptomics.

Pipeline	Setup	Samples	Algorithms	Outcomes	Ref
Data generation	10x Genomics scRNA-seq	Root cells	Droplet-based UMI sequencing	Single-cell transcriptome	(Zheng et al., 2017)
	10x Genomics snRNA-seq	Root tissues	Nuclear RNA profiling	Single-nucleus transcriptome	
	10x Visium spatial transcriptomics	Root sections	Spatial barcoding	Spatial gene expression	(Ståhl et al., 2016)
	MERFISH/seqFISH	Root tissues	Imaging-based hybridization	High-resolution spatial maps	(Chen et al., 2015; He and Pryhuber, 2025)
	10x Multiome	Root cells	RNA + ATAC co-assay	Multiomics datasets	(Ma et al., 2020)
Processing	FastQC	FASTQ	Quality control metrics	Read quality assessment	(Andersson et al., 2010)
	STAR	FASTQ	Alignment to plant genome	Mapped reads	(Dobin et al., 2013)
	Cell Ranger	10x data	UMI counting pipeline	Gene cell expression matrix	(Zheng et al., 2017)
Normalization	Seurat	Expression matrix	Filtering + normalization	Cleaned dataset	(Stuart et al., 2019)
	Scanpy	Expression matrix	Filtering + scaling	Preprocessed data	(Wolf et al., 2018)
Feature range & range reduction	Seurat	Expression matrix scRNA-seq	HVG selection, PCA Graph-based clustering	Reduced dimensions Root cell clusters	(Stuart et al., 2019)
Clustering of cells	Scanpy	Expression matrix scRNA-seq	PCA, UMAP Leiden/Louvain clustering	Visualization-ready embeddings Cell populations	
					(Wolf et al., 2018)
	SingleR	scRNA-seq	Correlation-based labeling	Cell identity assignment	(Aran et al., 2019)
Cells annotation	scmap-cell	scRNA-seq	KNN mapping	Projection to reference	(Kiselev et al., 2018)
	Garnett	scRNA-seq	Marker-based classification	Automated annotation	(Pliner et al., 2019)
Analysis & integration	Seurat	scRNA-seq	Statistical testing	Marker genes	(Hao et al., 2024b)
	Scanpy	scRNA-seq	Statistical testing	Cluster markers	(Wolf et al., 2018)
Data masking & combination	Harmony	Multi-sample	Soft clustering correction	Batch-corrected data	(Korsunsky et al., 2019)
	scVI	Multi-batch	Variational autoencoder	Integrated latent space	(Lopez et al., 2018)
	Scanorama	Multi-dataset	MNN + SVD	Dataset integration	(Hie et al., 2024)
	LIGER	Multi-omics	NMF	Joint factor analysis	(Welch et al., 2019)
Trajectory analysis	Monocle	Root development	Pseudotime inference	Developmental paths	(Trapnell et al., 2017)
	Slingshot	Lineage data	Curve fitting	Lineage structure	(Street et al., 2018)
	scVelo	Dynamic cells	RNA velocity	Future cell states	(Bergen et al., 2020)
Spatial transcriptomics	Tangram	scRNA + spatial	Probabilistic mapping	Cell localization in roots	(Biancalani et al., 2021)
	Cell2location	Spatial data	Bayesian inference	Cell-type abundance	(Kleshchevnikov et al., 2022)
	SPOTlight	Spatial data	NMF regression	Deconvolution	(Elosua-Bayes et al., 2021)
	RCTD	Spatial data	Poisson model	Cell proportion	(Cable et al., 2022)
	DestVI	Spatial data	Deep generative model	Spatial cell states	(Lopez et al., 2022)
Cell crosstalk identity	CellChat	scRNA-seq	Network inference	Signaling pathways	(Jin et al., 2021)
	CellPhoneDB	scRNA-seq	Ligand-receptor model	Interaction networks	(Efremova et al., 2020)
	NicheNet	Sender–receiver	Ligand-target modeling	Regulatory interactions	(Browaeys et al., 2020)

**Table 2:** Representative single-cell transcriptomic studies of plant root biology.

Plant	Aim of study	Tissue/cell	Key findings/highlights	Ref
Tea plant (*Camellia sinensis*)	Root specific metabolism	Roots tips	*CsLBD37*	(Lin et al., 2024).
*Medicago truncatula*	Nodule development	Lateral root	*STYLISH4*	(Pereira et al., 2024)
*Arabidopsis thaliana*	Callus induction	Roots	*CP23*, *GBF4*, *RTV1*, and *bHLH104*	(Yin et al., 2024)
*Arabidopsis thaliana*	root development and metabolism	Roots	*NRL27*	(Han et al., 2024)
*Arabidopsis thaliana*	Root tips function	Roots	*Shortroot-2* and *scarecrow-4*	(Shahan et al., 2022)
Sweet potato	Adventitious Root tip development	Seedling	*IbGATA4*	(Zhao et al., 2024)
Arabidopsis & Soybean	Root tips function	Roots	*PIF3-receptor kinase FER*	(Xu et al., 2024)
*Arabidopsis thaliana*	Root tips function	Root tips	*TMO5/LHW*	(Wendrich et al., 2020)
*Arabidopsis thaliana*	Cellular differentiation development	Root tips	QC related genes	(Ryu et al., 2019)
*Arabidopsis thaliana*	changes cell specific RNA expression	Roots	Cell specific gene’s dynamics	(Jean-Baptiste et al., 2019)
*Arabidopsis thaliana*	lateral root primordia	Root tips	*XPP, LRP, PPP, ARF7, ARF9*	(Gala et al., 2021)
*Arabidopsis thaliana*	Cell-Cell communication and chromatin effects on gene expression	Roots	100 markers including *AT1G61590, AT2G40160, AT2G48130, AT4G17215, AT5G18840* and *AT3G21670*	(Farmer et al., 2021)
*Arabidopsis thaliana*	Impact of osmotic stress in root tip	Roots	Candidate stress-related gene-linked *cis*-regulatory elements (gl-cCREs)	(Liu et al., 2024)
*Arabidopsis thaliana*	Brassinosteroids action root map	Roots	Brassinosteroids-responsive genes	(Graeff et al., 2021)
*Arabidopsis thaliana*	Brassinosteroids (*BRI1*) homeostasis assessment	Root tips	*BIR3*^EXT^-*BRI1*^INT^, *BRI1, BRL1, BRL2, and BRL3*	(Blanco-Touriñán et al., 2024)
*Arabidopsis thaliana*	mechanosensitive	Roots	receptor kinase–TF module (FER interacted with and stabilized *PIF3* to modulate the expression of mechanosensitive ion channel *PIEZO*	(Liu et al., 2023; Xu et al., 2024)
*Arabidopsis thaliana*	Alternative splicing and long intergenic noncoding RNA (lincRNA) regulation	Roots	Distinct isoforms of coding regions, intron retention and distinct transcriptome actors	(Li et al., 2016)
*Arabidopsis thaliana*	developmental regulatory programs	Seedlings	Developmental trajectories enable visualization of cell specification events scRNA-seq of the *scarecrow* mutant reveals a cell identity change occurs over time	(Shahan et al., 2022)
Cotton (*Gossypium arboreum*)	Salt stress	Lateral roots	*GaGH3*.*6*	(Li et al., 2024)
Rice (*Japonica* group cultivar Nipponbare (Nip) and *Indica* group cultivar 93-11)	Cell-type specification at transcriptome level in monocots root tips	Root tips	*LOC_Os07g43670*	(Liu et al., 2021)
*Zea mays*	Elucidation of *SHORT-ROOT (SHR)* signaling pathway	Root tips	*ZmSHR1, ZmSHR2*, and *ZmSHR2-h*	(Ortiz-Ramírez et al., 2021)
*Zea mays*	Transcriptional regulation network in response to Fungal invasion	Roots	*Fv*-responsive regulatory modules from 4049 differentially expressed genes (DEGs)	(Cao et al., 2023)
*Triticum aestivum*	Regulatory network	Roots	*TaSPL14* participates in vasculature development	(Zhang et al., 2023)
*Lotus japonicus*	Legume root development	Root tips	Insights to phytohormone and nodules	(Sun et al., 2023)
*Manihot esculenta*	Tuberous root		Cell types related to casparian strip	(Song et al., 2022)
*Solanum lycopersicum*	Transitioning of root system architecture	Roots	*LATERAL ORGAN BOUNDARIES DOMAIN (LBD)- Solyc09g066270*	(Omary et al., 2022)
*Medicago truncatula*	roots specific symbiotic relationship	Roots	*MtLIN, MtPUB2, MtENODL13, MtDMI2, MtCHK1/MtCRE1, MtRRA2, MtZPT2-1, MtDNF2, MtDMI3, MtRRB24, MtZPT2-1, MtZPT2-2, MtPIP1, MtCASTOR, MtARF2, MtARF8, MtDELLA1, MtDELLA2, MtRRB9, MtEIN3, MtBAK1, MtNLP1, MtCASTOR, MtNCR112, MtZPT2-1, MtZPT2-2, MtDNF2, MtNAC969, MtLIN, MtNF-YA1, and MtNPL*	(Cervantes-Pérez et al., 2022)
Soybean	nodule maturation	Seedlings	*GLYMA_02G004800*	(Liu et al., 2023)
Soybean	root and mature nodule	Seedlings	*GmFWL3*	(Cervantes-Pérez et al., 2024)
Rice (*Nipponbare*, *Zhenshan 97* and *Minghui 63*)	Identification of open chromatin regions	Roots	*OsHAK12, RCc3*	(Zhu et al., 2024)

### 2.2 Gene regulatory networks and hormonal control of cell fate

Single-cell RNA sequencing (scRNA-seq) resolves heterogeneous *in vivo* root GRNs and dynamic transcriptional landscapes through organ-scale cell atlas construction in model organisms such as *Arabidopsis thaliana* (Shahan et al., 2022). Lineage priming, cell fate determination, and tissue patterning are central to plant development, particularly during root organogenesis (Nolan and Shahan, 2023). Root growth is sustained through tightly regulated molecular networks coordinating cell proliferation, longevity, and tissue expansion (Lyu et al., 2025). These processes are coordinated through dynamic interactions among neighboring tissues, mechanical constraints, chromatin remodeling and transcriptional regulatory networks that collectively govern cell fate acquisition (Dorrity et al., 2021). The contribution of phytohormone-mediated GRNs to cell type specification remains unresolved (Zhu et al., 2025). Recent scRNA-seq advances have begun to elucidate the molecular pathways underlying previously unresolved aspects of root development. Transcriptional reprogramming of stem cells during root formation drives sequential cell type fate transitions, establishing a developmental continuum (Shahan et al., 2022; Cabrera et al., 2024). Each cell type exhibits a dynamic transcriptomic signature that is spatiotemporally regulated throughout development. The fate decisions are governed by spatiotemporal gradients, mediated through the fine-scale transcriptional reprogramming within the root meristem (Nobori et al., 2023; Nolan and Shahan, 2023). Such a complex control of root development is orchestrated through dynamic, hormone-regulated GRNs operating across heterogeneous cell populations (Oliva et al., 2022). Pseudotime analysis of scRNA-seq data resolved the transition from mitotic cycling to endoreduplication, in which endocycle entry coincides with reduced auxin signaling and declining *CYCB* expression (Zhang et al., 2019). Gibberellins, jasmonic acid (JA), abscisic acid (ABA), brassinosteroids (BR), cytokinin and ethylene additionally regulate cell division, elongation and differentiation (Lyu et al., 2025; Zhu et al., 2025). The antagonistic interaction between auxin and cytokinin defines the boundary between the meristematic and elongation zones, with regulatory roles attributed to *CYTOKININ RESPONSE FACTOR 2 (CRF2)* and *PIN2 PROMOTER BINDING PROTEIN 1 (PPP1)* (Zhao et al., 2024). Brassinosteroids (BR) signaling acts as a non-cell-autonomous regulator of epidermal cell fate, formative divisions, and cell elongation coordination in *Arabidopsis thaliana* root development (Jean-Baptiste et al., 2019; Xu et al., 2024). Graeff et al., 2021, constructed a single-cell transcriptomic map relating BR signaling to division plane orientation and to the timing of cell expansion, identifying the cortex and elongation zone as the principal sites of BR response, mediated in part by *Arabidopsis thaliana HOMEOBOX 7 (ATHB7)* and *GT-2-LIKE 1 (GTL1)* (Gala et al., 2021; Graeff et al., 2021). These transcription factors promote cell elongation through transcriptional activation of cell wall biosynthetic genes, including *CELLULOSE SYNTHASE (CESA), CELLULOSE SYNTHASE INTERACTIVE 1 (CSI1)*, E*xpansins* *(EXPs)* *and xyloglucan endotransglucosylase/hydrolases (XTHs)*. Metabolic pathways further modulate the cell cycle through auxin-coordinated signaling (Farmer et al., 2021). Evolutionarily conserved transcription factors *SHORTROOT (SHR), SCARECROW (SCR)*, and *PHABULOSA (PHB)* are required for radial tissue patterning and cell type specification (Sabatini et al., 2003; Zhao et al., 2024). The *miR165/166* restricts *PHB* transcript accumulation in the peripheral tissues and the QC, thereby maintaining stem cell identity (Carlsbecker et al., 2010; Miyashima et al., 2011). Cell-resolved mapping of root secondary metabolism, for example theanine biosynthesis in *Camellia sinensis*, has resolved metabolic networks restricted to particular root cell types (Lin et al., 2024). Combining scRNA-seq with other single-cell genomic assays therefore allows molecular and transcriptional change to be placed in spatial context. The *SUC2, SHR, SCR, PLETHORA (PLT)* and hormone-related genes including the *PIN* auxin efflux carriers serve as molecular markers (Wendrich et al., 2020) for tracing cell fate transitions across developmental, metabolic, and physiological contexts (Zhao et al., 2024). The evolutionary analysis of root developmental mechanisms in monocots and dicots identifying conserved regulators of root hair growth alongside anatomical divergences between *Oryza sativa* and *Arabidopsis thaliana* (Ryu et al., 2019). They also found considerable cell-type-specific heterogeneity of long non-coding RNAs (lncRNAs) at single-cell resolution, including cell-specific regulons like *HSFA1E* in the lateral root cap (Jean-Baptiste et al., 2019). The motif-informed network inference from single-cell expression data (MINI-EX) framework has been used to reconstruct transcriptional regulatory networks governing root development in both *Arabidopsis thaliana* and *Oryza sativa* (Ferrari et al., 2022). Single-cell studies of salinity stress implicate ABA and auxin in the control of root cell fate under stress: epidermal, xylem and endodermal cells display strong ABA-associated transcriptional signatures, while auxin distribution across root tissues tracks developmental regulation (Song et al., 2022; Li et al., 2024). PhytoMap, a plant-adapted *in situ* hybridization platform, permits simultaneous spatial visualization of up to 28 target genes across developmental stages and stress conditions (Nobori et al., 2023), placing transcriptional relationships within their local cellular niche. Integration of multiple scRNA-seq datasets extends such analysis to regulatory networks at broader biological scale (Stuart et al., 2019; Zhang et al., 2024). The scRNA-seq identifies the transcriptional changes, transcription factors and regulatory networks associated with cell state transitions (Serrano-Ron et al., 2021). Identifying the loci and regulatory factors activated during these transitions will further define the machinery driving meristem maintenance, proliferation, transition, elongation and differentiation (Han et al., 2023).

## 3 Single-cell transcriptomic dissection of root responses to environmental signals

Plants are exposed to biotic and abiotic stimuli that influence growth and development throughout the life cycle (Sun et al., 2023). Roots form the primary interface with the soil and adjust their development in response to environmental signals (Jean-Baptiste et al., 2019). Survival and productivity depend on the capacity of the root to respond to light, nutrient availability, water status and gravity (van Gelderen et al., 2018). These cues act on root development and elongation through cellular mechanisms that are themselves the expression of developmental plasticity in root cell populations. Lineage tracing demonstrates that meristematic cells can be reprogrammed to alternative fates according to positional and environmental cues (Cortleven et al., 2019). Dynamic regulation of root traits is central to crop performance and environmental adaptation, necessitating investigation of the molecular and cellular mechanisms underlying phenotypic variation (Uga et al., 2013). Dissecting transcriptional responses at single-cell resolution across environmental conditions has therefore become a priority, in order to assign cell-type-specific contributions at tissue scale (Ferrari et al., 2022). Single-cell transcriptomics resolves stress-responsive networks at cellular resolution (Han et al., 2023). Under heat stress, Arabidopsis root cells activate the canonical heat shock response, but do so unevenly: non-hair, phloem and columella cells show pronounced transcriptional responses, whereas other populations respond weakly (Jean-Baptiste et al., 2019). This heterogeneity indicates that the heat shock response is spatially partitioned within the root rather than uniform across it. Light acts both as an energy source and as a developmental cue (Omary et al., 2022). The transition from skotomorphogenesis to photomorphogenesis triggers extensive transcriptome reprogramming in adaptation to altered light conditions (Cervantes-Pérez et al., 2022). The scRNA-seq identified a root cap cell cluster associated with mechanosensing, in which *PHYTOCHROME-INTERACTING FACTOR 3 (PIF3)* is stabilized through interaction with the receptor kinase *FERONIA (FER)*, modulating expression of the mechanosensitive ion channel *PIEZO* in *Arabidopsis thaliana* (Xu et al., 2024). Transcriptional responses to changing light are correspondingly plastic in aerial tissue, whereas root cells remain comparatively stable, consistent with adaptation to a subterranean environment (Cervantes-Pérez et al., 2024). Nutrient signaling illustrates the same point. Shanks et al., 2024, showed that single-cell approaches resolve nitrogen sensing and its regulatory networks in a spatiotemporally defined manner. Sucrose, the principal product of photosynthesis, acts in the root both as a carbon source and as a signal coupling nutrient status to morphological adjustment (Shanks et al., 2024). Arabidopsis root scRNA-seq pseudo-time analysis revealed extensive transcriptional reprogramming of the endodermis in the presence and absence of sucrose (Li et al., 2024). These data show how regulatory systems redirect root cell developmental trajectories according to resource availability (Wang et al., 2021b). Soil salinity is a major constraint on growth and crop productivity. Single-cell transcriptomics of *Gossypium arboreum* root under high salinity identified auxin-responsive genes, including *GaGH3.6* as a mediators of salt tolerance through redox regulation (Li et al., 2024). Salinity also depletes cell populations in the xylem, cortex and endodermis, with consequent loss of root structural integrity. Moreover, the study revealed that genes broadly expressed in outer root cell layers including trichoblasts, atrichoblasts and cortical cells exhibit pronounced stress susceptibility (Song et al., 2022). Wang et al., 2021b characterized transcriptional responses to low nitrogen, high salinity and iron deficiency in rice (*Oryza sativa*) dimension of root adaptive biology is symbiosis with nitrogen-fixing bacteria, where single-cell profiling has resolved the early, spatially localized interactions between microbial symbionts and root cells (Wang et al., 2021b). Cortical cell reprogramming has been identified as a central step in nodule organogenesis by single-cell profiling of *Medicago truncatula* roots inoculated with Sinorhizobium meliloti, in which phytohormone-mediated regulation implicates transcription factors such as *STYLISH4* (Pereira et al., 2024). Comparable pathways recovered from soybean nodules indicate that these regulatory modules are partly conserved, and extend to systemic signaling and immunity (Liu et al., 2023). Serrano et al., 2024 reviewed, how single-cell sequencing is refining mechanistic accounts of both legume–rhizobia and mycorrhizal symbioses (Serrano et al., 2024). Identification of conserved differentially expressed genes (cDEGs) in single-cell data has further defined the regulatory programs governing root developmental plasticity (Hoang et al., 2020). Taken together, these studies describe how roots integrate environmental signals at the level of individual cell types. Their translation to crop performance remains to be demonstrated, since most datasets derive from controlled-environment seedlings rather than field-grown plants (Yang et al., 2025; Zhu et al., 2025).

## 4 Single-cell approaches to epigenetic regulation in the root

Epigenetic regulation governs the molecular processes that determine cellular identity and transcriptional activity, and is therefore central to plant development and stress resilience (Gaude et al., 2024). The scRNA-seq resolves cellular heterogeneity in transcript abundance (Qu et al., 2024), but chromatin accessibility, DNA and RNA methylation, histone modification and nucleosome positioning are not read out by transcript sequencing and require dedicated single-cell epigenomic assays. Relating these layers to transcriptional variation is what defines root developmental plasticity at the chromatin level (Karlova et al., 2021). These assays resolve transcription factor binding sites (TFBS) and the regulators governing root hair development within heterogeneous cell populations (Fang et al., 2024). They remain limited, however, by low per-cell coverage, which restricts confident assignment of accessibility to rare cell populations and requires aggregation across cells before regulatory inference. Jean-Baptiste et al., 2019, showed that heat shock induces extensive chromatin remodeling in *Arabidopsis thaliana* seedlings, with marked increases in accessibility across promoters and coding regions (Jean-Baptiste et al., 2019). Combining single-cell chromatin accessibility data with machine learning in rice yielded 117,176 open chromatin regions (OCRs), a regulatory resource for rice root biology. These analyses identified a root-specific SNP-containing enhancer at *OsHAK12*, which encodes a potassium transporter and a further element at *RCc3* associated with lateral root development which carries a distinct chromatin signature (Zhu et al., 2024). Yan et al., 2024, constructed a cell-type-specific cis-regulatory element (CRE) atlas across five grass species, identifying *H3K27me3* as a conserved repressive mark within root epigenomic regulation and locating *LATERAL ROOT DEVELOPMENT 3* within accessible chromatin regions that define root cell identity in *Sorghum bicolor* and *Oryza sativa* (Yan et al., 2024). Emerging methodologies, including uCoTarget and uCoTargetX, enable simultaneous mapping of histone modifications, transcription factor occupancy and multimodal epigenomic profiles (Xiong et al., 2024). Together these methods sharpen resolution of the mechanisms governing root development (Nobori, 2025; Zhang et al., 2025). Whether the cis-regulatory elements they recover can be used as breeding targets is not yet established: cell-type-resolved accessibility maps exist for only a small number of species, and functional validation of individual elements in crops remains scarce (Ke et al., 2025; Zhu et al., 2025).

## 5 scRNA-seq in conjunction with multi-omics

**Figure 3:**
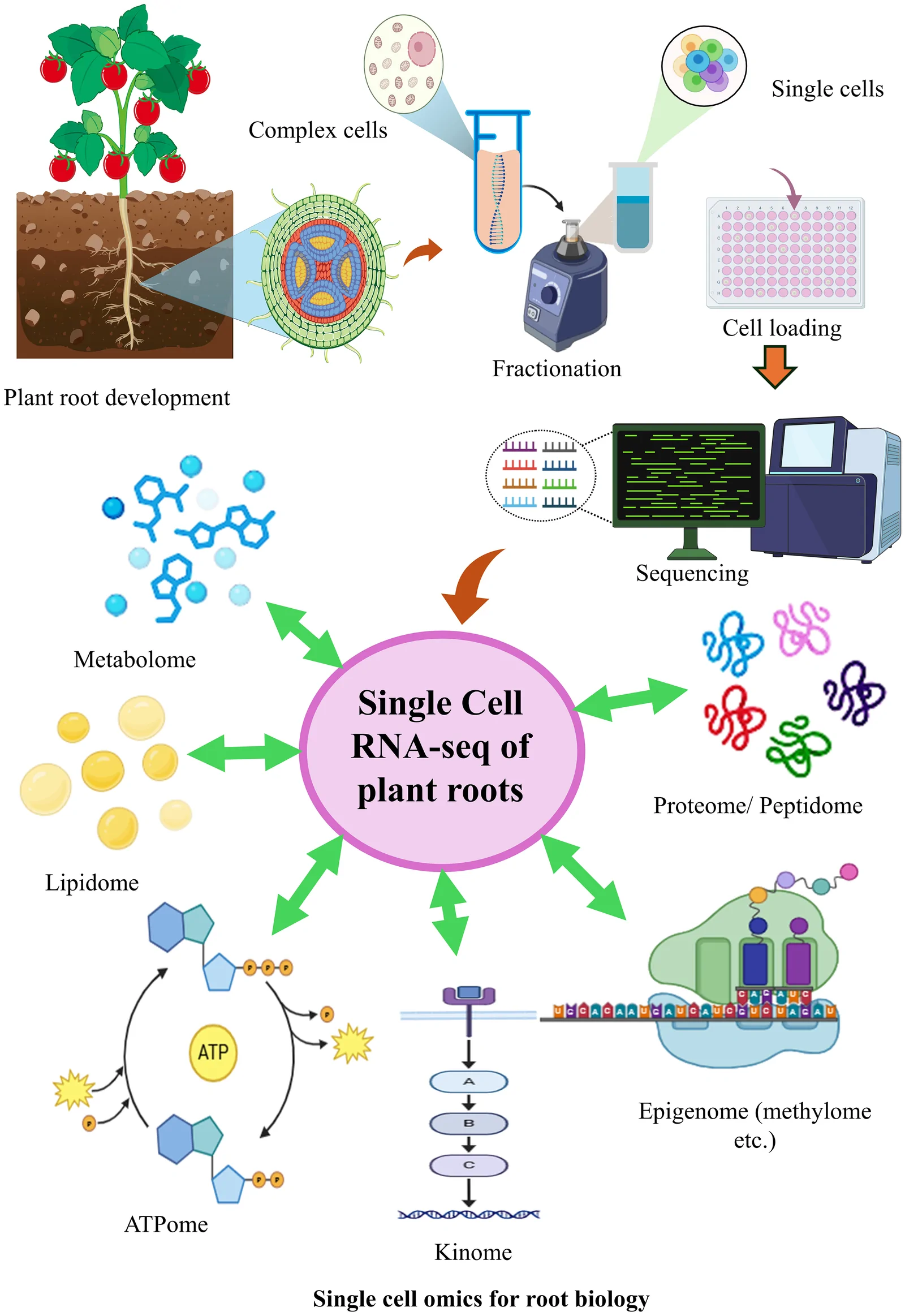
Single-cell multi-omics in plant root biology. scRNA-seq of root cells is integrated with epigenome, kinome, ATPome, lipidome, proteome and metabolome measurements to give a systems-level account of root development and environmental adaptation.

Combining single-cell sequencing with functional genomics allows the molecular networks regulating root development to be dissected systematically (Shahan et al., 2022). Cellular differentiation and tissue patterning are governed by layered control networks, and identifying their master transcription factors, signaling components and epigenetic regulators is what resolves that layering (Oliva et al., 2022). Single-cell transcriptomics resolves gene expression dynamics, while epigenomic, proteomic and metabolomic profiling addresses the regulatory basis of intercellular relationships and the spatiotemporal organization of the root (**Figure 3**) (Yu et al., 2023). Multi-omic profiling of the root tip under osmotic stress has identified osmotically responsive transcriptional regulators and established the contribution of chromatin accessibility to their control. Pseudotime trajectory analysis in the same system resolved stress-induced transcriptional reprogramming and identified gene-linked candidate cis-regulatory elements (gl-cCREs) mediating the response (Liu et al., 2024). The correlated chromatin and transcriptional signatures in root tissue under heat, drought and altered light using snRNA-seq and scATAC-seq resolving metabolic and developmental pathways together with their chromatin-level regulation (Liu et al., 2024; Mohan et al., 2024).

Montes et al., 2024, applied single-cell proteomics (SCP) to Arabidopsis roots, resolving 596 proteins enriched in the cortex and endodermis and thereby defining tissue-restricted proteomes (Montes et al., 2024). Coupling single-cell transcriptomics and metabolomics in Catharanthus roseus resolved cell-type-restricted expression across the monoterpene indole alkaloid pathway, showing that single-cell approaches can partition secondary metabolic pathways by cell type (Li et al., 2024). Reconstruction of intercellular signaling networks likewise provides mechanistic access to cell-type-specific responses to environmental perturbation (Dimitrov et al., 2024). Paired assays that couple transcript measurement to DNA methylation or to protein abundance in the same cell extend this resolution further. Nanobody-tethered transposition sequencing (NTT-seq) maps histone modifications and protein–DNA interactions simultaneously at single-cell resolution, resolving chromatin states within heterogeneous tissue (Stuart et al., 2023). Beyond chromatin, single-cell energy metabolism (ATPome) profiling (**Figure 3**) offers a route to the metabolic pathways underlying root establishment and maintenance (Bennett et al., 2020; Sun et al., 2024). Secreted peptides and small proteins produced during development and stress bind cognate receptors to initiate signaling cascades. Single-cell peptidomics could identify additional peptide regulators of root development in defined physiological states, although no plant root dataset of this kind has yet been reported. Kinases are principal transducers of environmental signals, and kinase profiling at single-cell level alongside single-cell transcriptomics would allow cell-type-specific biochemical pathways to be resolved (Kudo et al., 2018; Zhang et al., 2021a). Single-cell kinome profiling (**Figure 3**) may in turn separate responses by stimulus type, intensity and duration, and relate signaling state to the architectural plasticity of the root (Kudo et al., 2018). Lipids in turn determine membrane integrity, act in signaling and participate in energy metabolism. Notably, cholesterol constitutes approximately 10–20% of membrane sterols in cereal root cells (Valitova et al., 2016). Additionally, the lipids act as signaling mediators, interacting with receptors that regulate plant growth in response to surrounding stimuli (Zeng and Yao, 2022). Paired single-cell lipidomic and transcriptomic analysis could therefore inform cell fate transitions and differentiation events (Lin et al., 2024). Coupled to scRNA-seq, these single-cell multi-omic assays permit systems-level investigation of root developmental regulation (Yu et al., 2023). Their present limitation is throughput and cost: proteomic, metabolomic and lipidomic measurements at single-cell level currently cover far fewer cells and fewer analytes than transcript sequencing, so integration across layers is at present demonstrated on model species rather than on crops.

## 6 Machine learning and artificial intelligence in single-cell analysis

**Figure 4:**
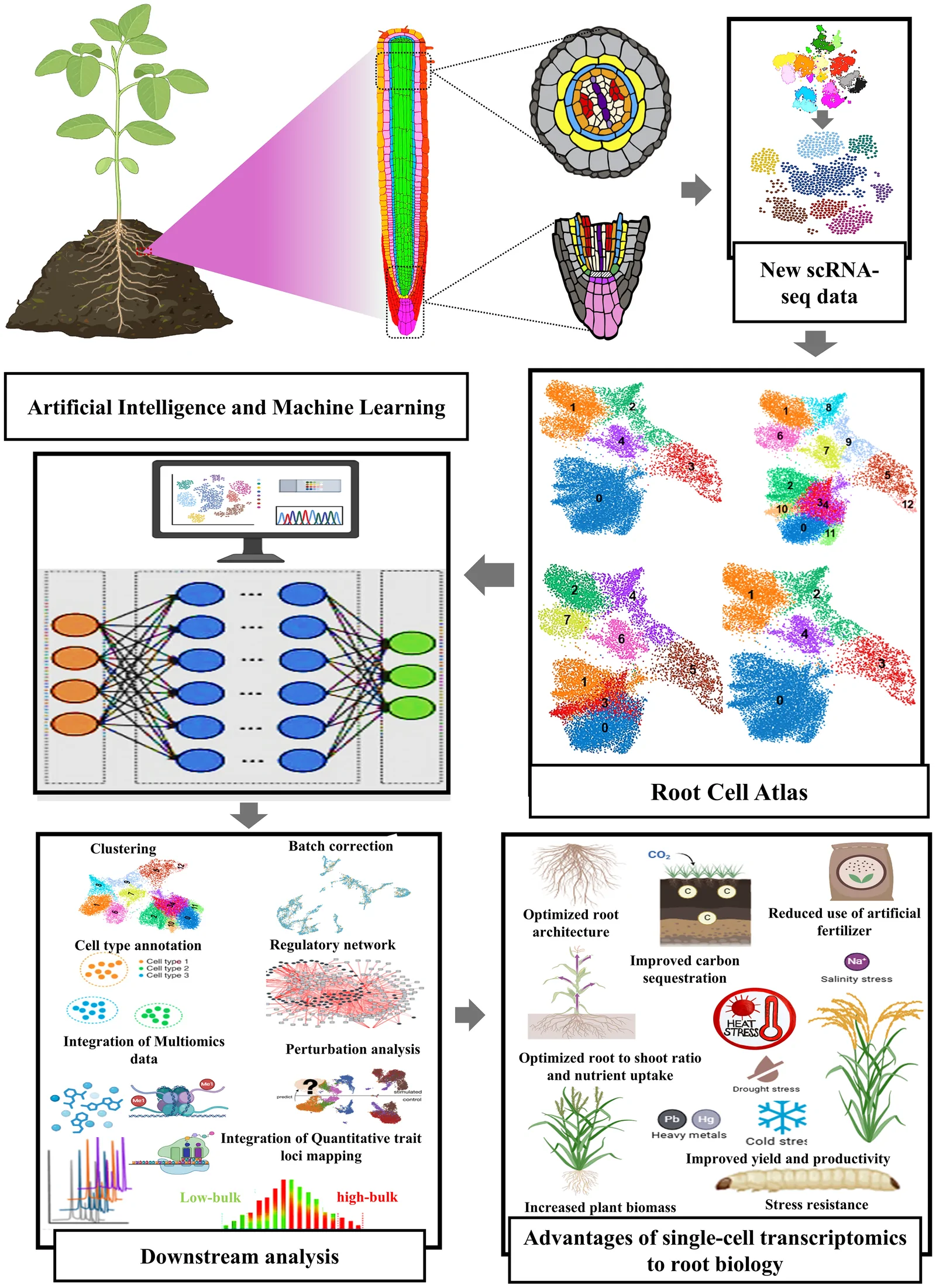
AI-driven single-cell transcriptomic framework for root biology. scRNA-seq data from root cell populations are analyzed through deep learning to generate root cell atlases, enabling cell-type annotation, multi-omics integration and GRN inference. These outputs support dissection of root trait genetics and the selection of candidate loci for stress tolerance and yield.

Deep learning architectures i.e., artificial neural networks (ANNs), convolutional neural networks (CNNs), generative adversarial networks (GANs) and graph neural networks (GNNs) are now applied directly to scRNA-seq matrices, and their combination with genomic data is reshaping analysis in functional genomics (**Figure 4**) (Wang et al., 2021a, 2023). Supervised classifiers including SingleR (Aran et al., 2019), scmap (Kiselev et al., 2018), Garnett (Pliner et al., 2019), CellAssign (Zhang T. Q. et al., 2019), scPred (Alquicira-Hernandez et al., 2019), singleCellNet (Tan and Cahan, 2019) and cellTypist (Domínguez Conde et al., 2022) annotate cells against reference datasets and marker gene sets. These rest on k-nearest neighbors, random forests, support vector machines and Bayesian models, and support high-throughput classification with transfer between datasets (Kharchenko, 2021). Deep architectures such as scDeepSort (Shao et al., 2021), scBERT (Yang et al., 2022) and TOSICA (Chen et al., 2023) report higher accuracy by modeling gene–gene dependencies, but both classes of method depend on well-annotated reference data and are correspondingly poor at recovering rare or previously undescribed cell populations i.e., a constraint that bears directly on crop species, for which no comprehensive reference atlas yet exists. Foundation models such as scGPT (Cui et al., 2024) and Geneformer (Zheng and Gao, 2023) address this through transfer learning, improving generalization across biological contexts (**Table 3**). Reportedly, both were pretrained on human data and their transferability to plant transcriptomes has not been systematically benchmarked. Applied to plant datasets, such models would permit genotype–phenotype relationships, including genotype-by-environment (G×E) interaction, to be examined at cellular level. Cao et al., 2023 used machine learning to construct cell-type-specific immune regulatory networks in maize roots responding to Fusarium verticillioides, recovering responsive regulatory modules from 4,049 differentially expressed genes. Such approaches reconstruct single-cell regulatory networks, nominate candidate biomarkers and yield predictive models of cell state transition (Cao et al., 2023). NRT-Predictor, an ensemble learning model, predicts cell-stage transitions in *Oryza sativa* root tissue and identifies the marker genes distinguishing developmental stages (Wang et al., 2023). Combining single-cell transcriptomic atlases with proteomic interactomes further resolves protein function at cell-type level (Li et al., 2024), although the resulting predictive models still require experimental validation. Generative pretrained models, whether single-cell specific such as scGPT (Cui et al., 2024) or general purpose such as the GPT series (Achiam et al., 2023; Singh et al., 2025), are being trialed in crop and horticultural genomics for the analysis of gene–gene interaction under stress. Iterative refinement against expanding datasets may resolve regulatory relationships not evident in individual experiments, providing a quantitative basis for predicting phenotypic response. This remains prospective: no published study has yet validated a generative model prediction of root stress phenotype against field data. Wang et al. (2024) introduced pseudo-genome divergence quantification (pgDQ), a framework for allele-specific expression (ASE) quantification in scRNA-seq data from polyploid genomes (Wang et al., 2024). It resolves subgenome-distinguishable expression clusters in polyploid species, including the root tip of wheat (*Triticum aestivum*), and quantifies the contribution of subgenome divergence to developmental processes. Quantifying that divergence cell by cell identifies the genes driving differentiation in polyploid backgrounds (Du et al., 2025). Integrating atlas-scale bulk transcriptomic data with transcription factor motif information allows a regulatory landscape to be assembled and master regulators of root development to be nominated. This shortens the path from candidate identification to functional testing of stress tolerance loci (Fan et al., 2025).

**Table 3:** Resources for scRNA-seq annotation using machine learning and AI.

Tools	Algorithms	Function	References
SingleR	Spearman correlation against reference profiles	Single-cell profiling with annotations of datasets	(Aran et al., 2019)
scmap-cell	k-nearest neighbors (KNN)	Matching query to datasets	(Kiselev et al., 2018)
Garnett	Elastic net regression	Marker-based cell type classification	(Pliner et al., 2019)
CellAssign	Bayesian model	Probabilistic marker-based cell type assignment	(Zhang A. W. et al., 2019)
scPred	Support vector machine	Cell type prediction from reference-projected features	(Alquicira-Hernandez et al., 2019)
singleCellNet	Random forest	Categorical classification	(Tan and Cahan, 2019)
CHETAH	Correlation-based hierarchical classification	Hierarchical classification with intermediate-node assignment	(de Kanter et al., 2019)
cellTypist	Logistic regression	Annotation by trained modeling	(Domínguez Conde et al., 2022)
CellBlast	Neural network model	Learns latent similarity space; enables cell querying like BLAST	(Cao et al., 2020)
sciBET	Multinomial distribution model	Multinomial-model supervised cell type identification	(Li et al., 2021)
scClassify	Weighted KNN	Ensemble class identity	(Lin et al., 2020)
scDeepSort	Graph neural network	Reference-free graph neural network cell type annotation	(Shao et al., 2021)
scBERT	Transformer	Gene–gene dependency modeling for cell type annotation	(Yang et al., 2022)
scAnnotate	Random forest	Ensemble annotation across reference datasets	(Ji et al., 2023)
TOSICA	Transformer	GO based identity and classification	(Chen et al., 2023)
scGPT	Pretrained transformer	Foundation model for single-cell data analysis and interpretation	(Cui et al., 2024)
Geneformer	Transformer	Transfer learning for gene network and cell state inference	(Zheng and Gao, 2023)
scFoundation	Large-scale deep learning	Foundation model pretrained on tens of millions of cells	(Hao et al., 2024a)
CellPLM	Pretrained language model	Cell language model using inter-cell context for annotation	(Wen et al., 2024)
scMulan	Deep learning	Multitask generative pretraining for cell type annotation	(Bian et al., 2024)

## 7 Conclusions

Resolving root organogenesis at cellular resolution advances mechanistic understanding of plant environmental adaptation and the design of resilient crops. This review has examined what single-cell RNA sequencing contributes to root developmental biology: resolution of cellular heterogeneity, definition of spatiotemporal signaling dynamics, and cell-type-specific stress response signatures. Coupling scRNA-seq to ATAC-seq and Ribo-seq allows cis-regulatory elements and transcription factors controlling root development and morphology to be identified. Multi-omic frameworks spanning transcriptomics, proteomics, metabolomics, epigenomics, post-translational modification and kinomics resolve root developmental programs at systems level. This would provide an understanding of complex gene regulatory networks (GRNs), epigenetic alterations, intercellular signaling influencing cell fate specification, root zonation and acquisition of tolerance to environmental stress factors. Generative and predictive machine learning models applied to these data will shorten the identification of master regulatory nodes governing root development and support inference of gene–gene and gene–TF-metabolite interaction. Three constraints qualify this outlook. Protoplast isolation imposes a documented bias against cell types with recalcitrant walls and induces a stress transcriptome of its own, which nuclear approaches only partly circumvent. Transcript dropout leaves single-cell matrices sparse, so low-abundance regulators are systematically under-detected. The reference atlases remain concentrated in Arabidopsis and rice, limiting the transferability of both annotation and network inference to the crops for which the approach is ultimately intended. Addressing these three constraints, rather than adding further assay modalities, is the immediate requirement if single-cell root biology is to contribute to resilience and productivity under climate change.
